# 

**Published:** 1877-05

**Authors:** 


### CONTENTS:

ARTICLE I.—The American Academy of Dental Surgery ........ 1

ARTICLE II.—Death of Dr. Chas. A. Jourdan from Effects of Chloroform ........ 6

ARTICLE III—The Susquehanna Dental Association ........ 7

ARTICLE IV.—Vulcanite Litigation ........ 8

ARTICLE V.—Concerning the Causes of Failures in the use of Cohesive Gold Foil; the Mallet, Etc ........ 22

ARTICLE VI.—On Congestion of the Pulp. By Prof. Geo. T. Barker, D. D. S. ........ 31

ARTICLE VII.—The Use of Salicylic Acid in the Household. By Dr. Von Heyden, 35

ARTICLE VIII.—Adenoma of Soft Palate ........ 37

ARTICLE IX—Atmospheric Anæsthesia. By M. J. Eley, M. D ........ 39

ARTICLE X.—Possible Dangers from Salicylic Acid ........ 39

### EDITORIAL, ETC.

Comparative Anatomy ........ 40

### BIBLIOGRAPHICAL.

Taft's Operative Dentistry, Third Edition ........ 44

### OBITUARY.

Dr. William D. Jenks ........ 45

### MONTHLY SUMMARY.

Dislocation of the Malar Bone ........ 46
A Secret Pile Remedy ........ 46
Local Action of Astringents upon the Vessels ........ 47
A Young Mother ........ 48
Therapeutic Action of Carbolized Camphor ........ 48

